# Multipotent stromal cells/mesenchymal stem cells and fibroblasts combine to minimize skin hypertrophic scarring

**DOI:** 10.1186/s13287-017-0644-9

**Published:** 2017-09-05

**Authors:** Cecelia C. Yates, Melanie Rodrigues, Austin Nuschke, Zariel I Johnson, Diana Whaley, Donna Stolz, Joseph Newsome, Alan Wells

**Affiliations:** 1 Department of Pathology, University of Pittsburgh, 3550 Terrace St., Scaife Hall, S-713, Pittsburgh, PA 15261 USA; 2 Department of Cell Biology, University of Pittsburgh School of Medicine, Pittsburgh, PA USA; 3 Department of Health Promotion and Development, University of Pittsburgh School of Nursing, Pittsburgh, PA USA; 4 Pittsburgh VAMC, Pittsburgh, PA USA; 5 McGowan Institute of Regenerative Medicine, Pittsburgh, PA USA; 6 Department of Plastic Surgery, Stanford University, Stanford, CA USA; 7 University of Pittsburgh, School of Nursing, 3500 Victoria Street, Victoria Bldg. 458A, Pittsburgh, PA 15261 USA

**Keywords:** Mesenchymal stromal cells, Fibroblast, Tenascin-C, Extracellular matrix, Cell therapy, Collagen and epidermal–dermal communication

## Abstract

**Background:**

Transplantation of mesenchymal stem cells (MSC) has been proposed to improve wound healing. However, as these cells only transiently survive in the implantation site, the mechanisms underlying this beneficial healing response are associated with restorative paracrine effects of MSC matricellular factors on resident stromal cells. However, this requires that the recipient has a robust reservoir of viable cells. Here, we examine the influence of MSCs on the behavior of cotransplanted fibroblasts, in a manner to provide augmented cellular reserve to debilitated individuals, specifically focusing on matrix remodeling following in-vivo wounding.

**Methods:**

Using a Hylan-A dermal filler hydrogel containing collagen I and tenascin-C for delivery and increased survival of transplanted cells, we find that cotransplantation of MSCs with fibroblasts reduces scarring.

**Results:**

Transplanted xenogeneic MSCs augmented fibroblast proliferation, migration, and extracellular matrix deposition critical for wound closure, and reduced inflammation following wounding. MSCs also corrected matrix remodeling by CXCR3-deficient fibroblasts which otherwise led to hypertrophic scarring. This effect was superior to MSC or fibroblast transplantation alone.

**Conclusions:**

Taken together, these data suggest that MSCs, even if eventually rejected, transplanted with fibroblasts normalize matrix regeneration during healing. The current study provides insight into cellular therapies as a viable method for antifibrotic treatment and demonstrates that even transiently engrafted cells can have a long-term impact via matrix modulation and education of other tissue cells.

## Background

Wound healing consists of a well-orchestrated series of time-dependent, overlapping events of coagulation, inflammation, new tissue formation, and resolution. These culminate in the repair of the collagen-rich dermis and the stratified epidermis [1]. During tissue formation fibroblasts, having migrated from the wound margins, deposit extracellular matrix (ECM) to form a granulation tissue that is invaded by newly formed vasculature. Upon resolution, the fibroblasts contract to draw the ends of the wound together, and replace the provisional prosynthetic matrix with a quiescent collagen I-rich tissue. The organization and remodeling of newly deposited ECM play a major role in determining the tensile strength of the healed wound [2]. Conversely, prolonged deposition of an immature ECM leads to hypertrophic fibrosis and scarring [3–5].

Fibroblast centrality in wound healing, results in them increasingly being tested in early clinical trials in the treatment of acute burn wounds, chronic wounds in diabetic and older people, and genetic conditions such as epidermolysis bullosa [6–8]. Another more widely tested source for cell therapy is the mesenchymal stem cell/multipotent stromal cell/marrow stromal cell (MSC) population obtained from expanding adherent mononuclear bone marrow cells in vitro. MSCs have been extensively tested for enhancing healing in several tissue systems, including the skin [9–12]. However, preclinical studies aiming for tissue generation have been thwarted by very low homing efficacy, poor long-term engraftment, and a limited capacity for differentiation of MSCs once transplanted in vivo [13, 14]. This has led to the focus of MSC therapy shifting from replacement to the trophic (‘cytokine factories’), and possibly immunological effects of these cells [15, 16]. This move from a cell source to modulator of repair has enabled the field to consider transient allogeneic transplantation rather than autologous cells, particularly for debilitated patients with limited or defective stem cell reserves. Following transplantation in preclinical cutaneous models, MSCs increase neovascularization, enhance matrix deposition, alleviate inflammation, and attenuate fibrosis through the release of growth factors during their sacrificial period of survival [17]. However, the effectiveness of transplanting either of these cell types on wound healing has been less than anticipated due in part to the limited persistence in the wound bed and intrinsic functioning that is dysregulated from the orchestrated progression of physiologic healing.

Here, we tested the hypothesis that these two cell types would act synergistically to improve the quality of healing by communicating to appropriately educate the native wound microenvironment. A combined MSC + fibroblast transplant was assessed for its ability to suppress hypertrophic scarring in a mouse wound model. Since MSCs have a low survival rate in vivo, we delivered the MSCs in a matrix containing collagen-I and tenascin-C. Collagen-I is a cell adhesive and suppressive component of the dermis; tenascin-C, on the other hand, is an anti-adhesive matricellular component upregulated during the healing response. We have reported previously that tenascin-C binds, via its EGF-like repeats, to the epidermal growth factor receptor on MSCs, and thereby increases their survival when implanted in vivo [18]. Taking this approach forward, we transplanted sheets of MSCs which had been expanded on the pro-regenerative prosurvival collagen–tenascin-C matrix along with equal numbers of fibroblasts within a Hylan-Polymer-A dermal filler onto excisional wounds. We used our recently developed wound-scarring model based on the absence of wound resolution through the CXCR3 signaling axis; these mice develop sterile chronic inflammation and hypertrophic scars [19]. The transplantation of MSCs and fibroblasts together with this matrix signaling system, which modulates migration and differentiation of stromal cells during healing, corrects the scarring defect. This study provides insight into conceptual therapies of cell transplantation.

## Methods

### Animals

C57BL/6J wild-type and CXCR3 expression-abrogated mice [20] were bred in-house and used at 7–10 weeks of age. FVB wild-type and CXCR3-deficient mice on a FVB background were also tested for reproducibility and rigor. All offspring were genotyped by PCR before use. As controls, age-matched C57BL/6 J mice were obtained from Jackson Laboratories (Bar Harbor, ME, USA).

### Fibroblast harvest and culture

The donor fibroblast cells were cultured according to existing protocols [21]. Cells were derived from neonatal mice (2–4 days old). Fibroblasts were harvested from primary culture after 2 days and either subcultured immediately or cryopreserved.

### MSC culture and MSC–fibroblast construct preparation

hTERT (imMSCs) were obtained from Junya Toguchida’s laboratory (Kyoto University, Japan). Cell culture dishes were coated for 16 hours at 37 °C with 1 μg/cm^2^ Col I, or 1 μg/cm^2^ Col I and 1 μg/cm^2^ TNC diluted in phosphate-buffered saline (PBS). Deposition of TNC on the surfaces was confirmed by immunofluorescence.

### In-vivo excisional wound model and xenograft transplant

Male and female mice (7–10 weeks old, weighing approximately 25–35 g) were anesthetized with an intraperitoneal injection containing ketamine (75 mg/kg) and xylazine (5 mg/kg). Cells were mixed and added into an 8-mm punch full-thickness wound to reduce contraction, using a syringe with hyaluronic acid (HA) noncross-linked polymer (Hylan A gel; Genzyme Biosurgery), until a homogeneous mixture was obtained containing approximately 1 × 10^6^ cells/ml. A 1-ml syringe with a 24-gauge needle was then used to xenotransplant ~50 μl of the HA–Col–TNC cell (MSC and/or fibroblast) mixture to each wound. Control wounds received HA only, or HA-MSC only, with no Col I–TNC.

### Histological assessment

Histopathological examination of mouse tissues was performed blinded by a veterinary pathologist using a validated scoring system described previously [22]. Qualitative assessments were made concerning aspects of dermal and epidermal maturation, inflammation, and granulation tissue. The samples were scored on a scale of 0–4 for epidermal maturation (0, no migration; 1, partial migration; 2, complete migration with partial keratinization; 3, complete keratinization; and 4, normal epidermis), dermal maturation (0, no healing; 1, inflammatory infiltrate; 2, granulation tissue present—fibroplasias and angiogenesis; 3, collagen deposition replacing granulation tissue > 50%; and 4, complete healing), and inflammation (0, abundant; 1, moderate; 2, mild; 3, slight; 4, none). Fibroblast infiltration and reactivity in the wounded area was determined by scoring the maturity of the fibroblast from most reactive to normal on a scale of 1–4.

### Immunohistochemistry

Skin from the excisional model was harvested at 3, 7, 21, and 30 days following wounding. These wound biopsies were fixed in 10% buffered formalin, processed, and embedded in paraffin blocks using standard protocols [23]. Tissue sections (5 μm) were stained with hematoxylin and eosin (H&E) and analyzed for general tissue and cellular morphology. Masson’s trichrome and Picrosirius Red staining using MetaMorph software analysis evaluated collagen deposits and collagen alignment [24]. Sections were stained using immunohistochemistry for immunohistochemically for tenascin-C (1:100, ab137508; Abcam), fibronectin (1:100, ab2413; Abcam), and CD3 (1:250, ab5690; Abcam).

### Dermal maturation assessment

A boarded veterinary pathologist, who was blinded to all treatments, performed histopathological examination of mouse tissues. Qualitative assessments were made concerning aspects of dermal and epidermal maturation, inflammation, and granulation tissue [19].

#### Quantitative real-time RT-PCR

RNA was isolated and reverse transcribed with a High Capacity cDNA Reverse Transcription Kit (Applied Biosystems, Rotkreuz, Switzerland). TaqMan real-time PCR primer/probe mixtures for mouse fibronectin (Mm01256734_m1), mouse tenascin-C (Mm00495662_ m1), mouse type I collagen (α-1 chain; COL1A1; Mm00801666_g1), and mouse glyceraldehyde 3-phosphate dehydrogenase (GAPDH; Mm99999915_g1) as well as TaqMan Universal Master Mix were purchased from Applied Biosystems. The primers and Taqman probes were designed using Primer Express software (Applied Biosystems, Foster City, CA, USA). Forward and reverse primers were purchased from Integrated DNA Technologies (Coralville, IA, USA) and fluoro-coupled Taqman probes were purchased from Applied Biosystems. The reverse transcription (RT) reaction (using reverse primer) and subsequent real-time PCR assays were performed using the same conditions as described previously [21]. For every experimental condition, reverse-transcribed cDNA (6 ng) was amplified for each of the three genes on an ABI Prism 7900 real-time PCR cycler (Applied Biosystems) using the comparative critical cycle (Ct) method and GAPDH as the endogenous control. Using the comparative critical cycle (Ct) method and using GAPDH as the endogenous control. Results shown are the synthesis of at least three independent determinations. Data were analyzed using 7900 HT SDS software version 2.1 provided by Applied Biosystems.

### In-vitro survival assays

Fluorochrome inhibitor of apoptosis (FLICA) is a cell-permeable and noncytotoxic inhibitor of caspase-3 bound to sulforhodamine, which emits red fluorescence when bound to active caspase-3. Lab-tek eight-chamber slides were coated for 24 hours with 1 μg/cm^2^ Col I, or 1 μg/cm^2^ Col I and 1 μg/cm^2^ TNC diluted in PBS, or left uncoated as described previously. The slides were counterstained with 4,6-diamidino-2-phenylindole (blue) ( Dapi) (Vector Laboratories, Burlingame, CA, USA) [18].

The in-situ cell death detection kit (fluorescein) (11 684 795 001) from Roche was used for the TUNEL assay as described previously [19].

### Cell starvation

To test for serum starvation, 0.1% and 0% fetal bovine serum (FBS) in Dulbeco’s modified Eagle’s medium (DMEM) was used to mimic an avascular wound. To test for glucose starvation, 0.5 g/L and 0.1 g/L in DMEM with no serum, which represent hypoglycemic and sever hypoglycemic conditions respectively, were used as an additional model for nutrient deprivation and mimicry of stressful environment. The Annexin V-Cy5 Apoptosis Detection Kit (K103-25) was used.

### Cytotoxicity and metabolic assays

Cell metabolic activity was assessed using a Vybrant Cell Metabolic Assay Kit (Invitrogen). The experiment was conducted as already described with six replicates/treatment and measurements were assessed at 24, 48, 72, and 120 hours.

### Analysis of explants for immune cell infiltration

Twelve-millimeter punch biopsies of wounds and 12-mm punch biopsies of unwounded regions were isolated for quantification of MSC engraftment in wounded skin at days 3, 7, 14, and 30. Tissue was minced and incubated in 0.5 mg/ml Liberase-TL (Roche Applied Science). Immune markers CXCR3 CD183 (562152), CD3 (552774), and CD11b (557672), antibodies (BD Biosciences), and F4/80 (123116; BioLegend) were used. Appropriate were used as controls. Flow cytometry was performed on an LSR II Cytometer (BD Biosciences) and subsequently analyzed using FlowJo digital FACS software (Tree Star, Inc.).

### In-vitro ECM deposition assays

MSC–fibroblast transwell cocultures were used with a 0.4-μm pore size and a diameter of 12 mm for 12-well plates (Costar Corporation) with or without tenascin-C coating. The inserts containing the MSCs were transferred onto 12-well dishes with the fibroblasts. The MSCs were then stimulated with or without epidermal growth factor (EGF; 10 nmol/L) and/or anti-CXCL11 (3 μg/ml) for 24 hours.

### Statistical analysis

Results are expressed as mean ± SEM with all individual experiments performed in triplicate. Statistical differences between groups were determined by the Student’s *t* test or one-way ANOVAs using GraphPad Prism 6 software. Statistical difference between individual groups or treatments was determined by post-hoc analysis using Bonferroni correction. Additionally, paired analyses were performed between all groups; the number of animals per group was determined a priori based on a 90% confidence of finding a 30% difference between the groups. Comparisons over time were performed by analysis of variance. Significance was claimed for *p* < 0.05.

## Results

### MSC augmentation enhances survival and migration of fibroblasts

**Fig. 1:**
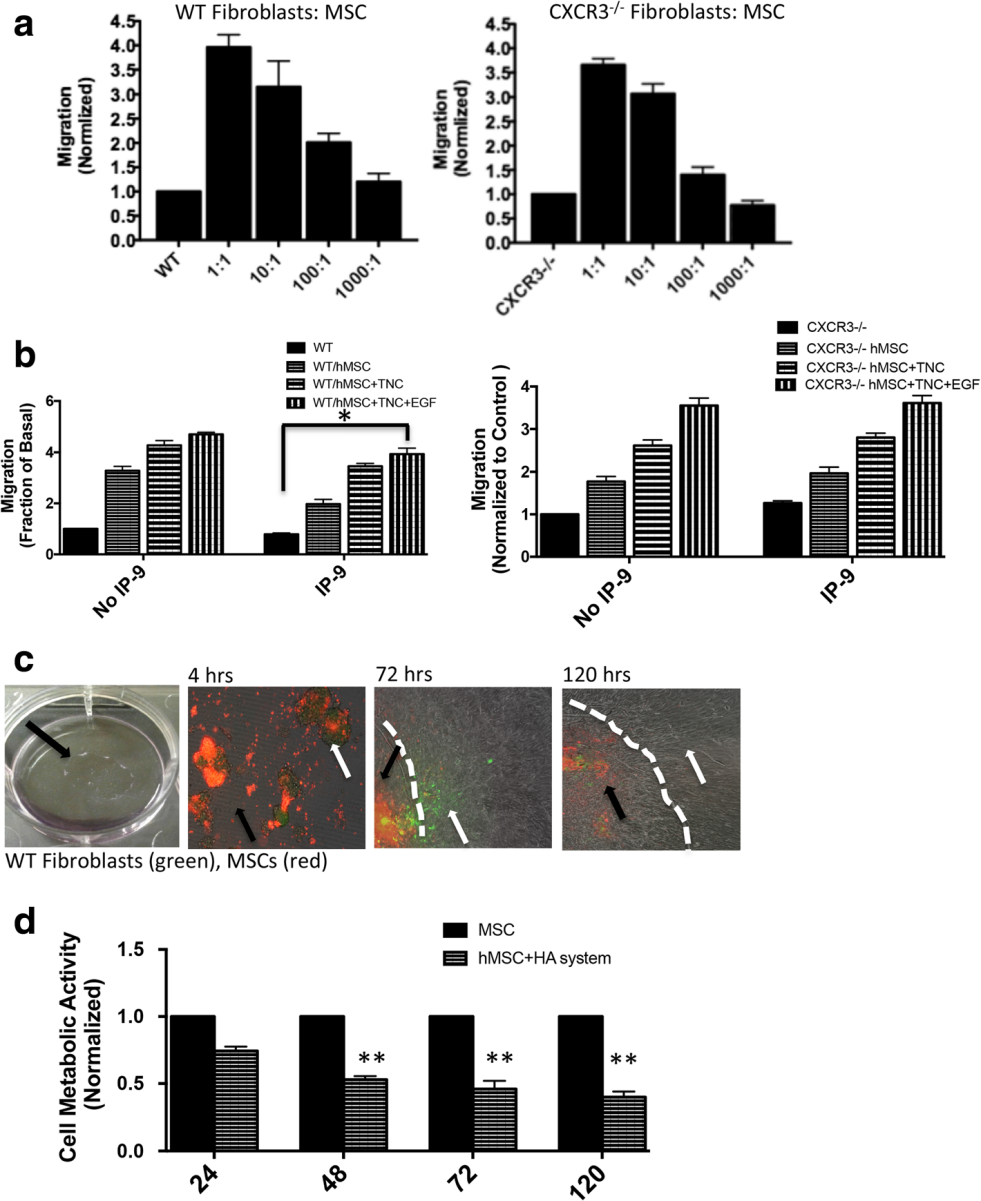
Matrix-bound MSCs improve fibroblast function. **a** Two-dimensional in-vitro “wound healing” assay, fibroblasts from mice (wild-type and CXCR3^–/–^) and MSCs fluorescently labeled and plated at varying ratios in 12-well plates. A 1 mm-wide “scratch wound” was made in the in monolayer using a rubber policeman and the migration into the “wound” determined at 24 hours (normalized to fibroblasts alone). **b** Two-dimensional in-vitro transwell “wound healing” assay, fibroblasts (bottom) and MSCs with/out treatment (top) seeded. The cells then incubated in 0.5% dialyzed DMEM alone with no treatment (NT) containing EGF (10 nmol/L) or in the presence of IP-9 (25 ng/ml). Fibroblasts in the bottom chamber assessed in an in-vitro “wound healing” assay, as in **a. c** Fluorescently labeled MSCs (red; black arrow) and fibroblasts (green; white arrow) plated in matrix gel plugs and cell outgrowth monitored for 5 days (white dotted line delineates HA gel edge). **d** Cell proliferation via Vybrant Cell Metabolic Assay comparing MSCs in the hydrogel matrix system versus the tissue cultured plastic. Marked and equivalent reductions in metabolic activity responses appreciated at 48, 72, and 120 hours. *p < 0.05 and **p < 0.01, compared to control. Data and images from a representative experiment of three with *n* = 3 per experiment per condition, each with a different cell isolate. Original magnifications, ×100. EGF epidermal growth factor, MSC mesenchymal stem cells, TNC tenascin-C, WT wild type (Color figure online)

The first step was to validate our hydrogel matrix system as a delivery vehicle for MSC + fibroblast treatment for increased survival, and as a ‘schoolhouse’ to educate fibroblasts to remodel the wound bed. We evaluated in vitro the MSC + fibroblast coculture cell morphology, viability, proliferation, and migration (with and without tenascin-C incorporated within the hydrogel system) in response to growth factor and chemokine stimulation. To test the effect of MSCs on wild-type or CXCR3-deficient fibroblast motility, cells were plated at varying ratios of MSCs to fibroblasts. At high ratios of MSCs to fibroblasts (1:1 and 1:10) the motility of both wild-type and CXCR3-deficient fibroblasts was increased (Fig. 1a).

CXCR3 ligands limit fibroblast motility [25, 26] through protein kinase A (PKA) phosphorylation and inhibition of *m*-calpain. We aimed to test the ability of MSCs to attenuate this chemokine inhibition. A transwell coculture wound assay [19, 27] was used in which MSCs in the collagen–tenascin-C incorporated hydrogel matrix system were seeded in the top insert and stimulated with CXCR3 ligand IP-9, and fibroblasts were seeded in the bottom wells. We found that wild-type and CXCR3^–/–^ fibroblasts migrated in response to EGF stimulation. Wild-type but not CXCR3^–/–^ fibroblast motility was limited by IP-9 when plated alone, as expected. However, the TNC and TNC + EGF treatment of the MSCs in the upper well overcame the inhibitory effect of IP-9 on fibroblast motility (Fig. 1b). These data suggest the effectiveness of MSC paracrine signaling in altering fibroblast responses even in the face of a key negative regulator of fibroblast immigration.

Enhanced migration and invasiveness was conferred on the fibroblasts by live cell tracking of both MSCs (red) and wild-type or CXCR3^–/–^ fibroblasts (green, CFSE a proliferation tracker) that were encapsulated in the tenascin-C-incorporated hydrogel matrix system and plated on a tissue culture plate. Cells were monitored for 5 days for outgrowth. At day 3, the majority of the MSCs (red) exhibited a rounded morphology and clustered inside the hydrogel while the fibroblasts (green, CFSE) began to migrate and proliferate out of the hydrogel matrix (Fig. 1c). MSCs within the hydrogel matrix appear to be in a quiescent state suggesting that the metabolic activity is lower in the hydrogel matrix system (Fig. 1c). Indeed, there was a persistent and marked reduction in cell metabolic activity of MSCs when plated in the hydrogel system compared to tissue culture plastic (Fig. 1d). These data coupled with the low cytotoxicity and proliferation of MSCs within the first 5 days suggest the hydrogel matrix system can serve as a functional microenvironment for MSC-stimulated fibroblast motility.

### MSCs in the collagen–tenascin-C matrix provided for enhanced fibroblast survival

**Fig. 2:**
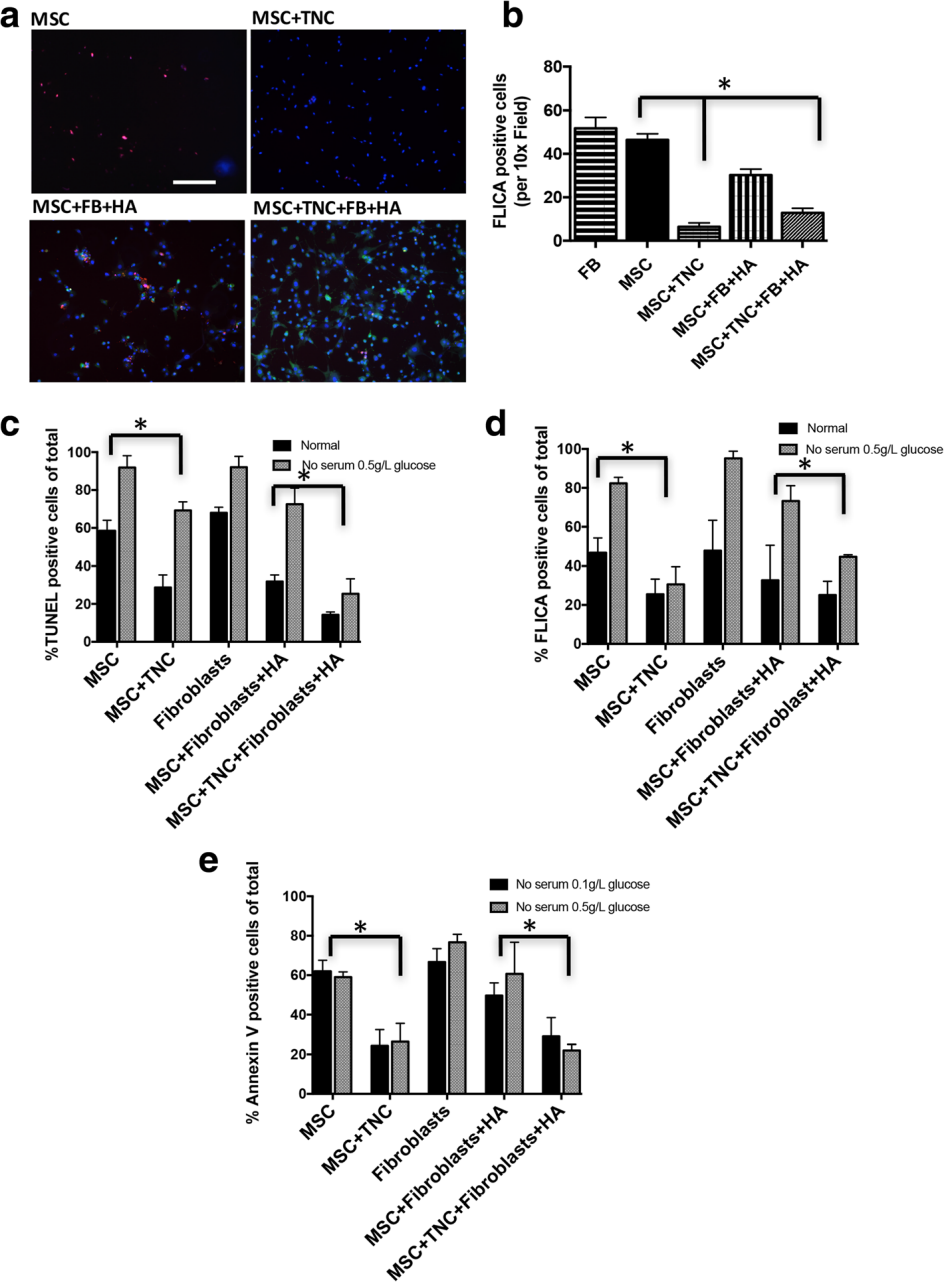
MSCs plated on a matrix provide a survival advantage for fibroblasts. **a** MSCs ± fibroblasts were plated with or without tenascin-C matrix on indicated surfaces and then subjected to a 12-hour treatment with FasL. Fluorochrome inhibitor of caspase activity (FLICA red)-stained MSCs and fibroblasts (tracked green) were visualized to monitor apoptosis. Counterstaining is shown in blue with DAPI. **b** Quantitative analysis using MetaMorph analysis of positive staining. Apoptosis was assessed in comprehensive cocultures in normal media or in no serum with 0.5 or 0.1 g/L glucose for MSC and fibroblast growth alone and with or without tenascin-C, and HA gel. Apoptosis was measured by TUNEL staining (**c**), FLICA positivity (**d**), or Annexin V positivity (**e**), respectively. *p < 0.05, **p < 0.01, compared to control. Data and images from a representative experiment of three trials, with at least *n* = 3 per experiment per condition, each with a different cell isolate. Original magnifications, ×100. FB fibroblasts, FLICA fluorescent inhibitor of caspase-3 activity, HA hyaluronic acid, MSC mesenchymal stem cells, TNC tenascin-C (Color figure online)

We have shown previously that tenascin-C protects MSCs from cell death induced by Fas [18]. Herein, we tested whether MSCs grown on tenascin-C can provide a survival advantage and reduce cell death in cotransplanted fibroblasts. In a direct coculture, MSCs and fibroblasts were grown on tenascin-C and subjected to FasL treatment to induce caspase-3 activation; fibroblasts were distinguished from MSCs by labeling with green cell tracker. We found that when MSC + fibroblast cocultures were on collagen–tenascin-C, the fibroblasts were basically protected from induced cell death as determined by fluorescent inhibitor of caspase-3 activity (FLICA, red) staining (Fig. 2a, b).

Another challenge transplanted cells experience is the low nutrient and hypoglycemic environment that can induce apoptosis [28]. We have reported that TNC is protective against death signals to increase MSC transplantation survival [13, 14]. To test for the survival of the transplanted cells in these conditions, and the protective effect of TNC combined with HA-gel, MSCs and fibroblasts were grown alone and/or in cocultures in normal media or in no serum with 0.5 or 0.1 g/L glucose with or with TNC and/or HA gel. Apoptosis was measured and detected by TUNEL to assess late-stage, caspase-3 activation after FasL treatment by FLICA, and early–mid-stage apoptosis by assessing the expression of phosphatidylserine (PS) on the cell surface with Annexin V (Fig. 2c–e). In all three assessments, the same overall trend was observed: there was a clear protective effect of the hydrogel matrix for fibroblasts in the coculture in a stressful environment of no-serum low-glucose conditions. HA gel did not prevent the TNC prosurvival effect of MSCs and fibroblast cells.

### MSCs alter dermal fibroblast matricrine signal expression

MSCs have been shown to influence other cells in the local environment and enhance tissue repair. MSCs increase production of several ECM proteins, which is thought to alter gene expression in dermal fibroblasts during repair. We have published previously that CXCR3^–/–^ fibroblasts delayed and altered production and remodeling of the ECM in vitro and in vivo [19]. To test the effect of MSCs on both normal (wild-type) and CXCR3^–/–^-impaired dermal fibroblasts, we focused on mRNA expression of ECM homeostasis genes because of the essential function in response to tissue injury (Table 1). Indirect transwell coculture of the wild-type fibroblasts with MSCs for 36 hours significantly upregulated expression of collagen type III (Col3a1) and matrix metallopeptidase 9 (Mmp9). In contrast, CXCR3^–/–^ functionally impaired fibroblasts significantly upregulated fibronectin (Fn1), tenascin C (Tnc), and collagen III (Col3a1) when cocultured with MSCs. These data suggest that matricrine signaling from MSCs may rescue impaired cellular function by altering fibroblast gene expression.

**Table 1:** Effect of mesenchymal stem cells on wild-type and CXCR3^–/–^ fibroblast expression of genes encoding extracellular matrix molecules

Gene symbol	GenBank™ accession number	Common name	Fold change + MSC/control (WT)	*p* value	Fold change + MSC/control (KO)	*p* value
*Fn1*	NC_000067	Fibronectin 1	1.91	0.0524	3.21	0.0317
*Tnc*	NC_000070	Tenascin C	1.33	0.0989	2.36	0.0497
*Lama1*	NC_000083	Laminin, alpha 1	0.89	0.5424	1.34	0.0605
*Col3a1*	NC_000067	Procollagen, type III, alpha 1	2.67	0.04	2.85	0.035
*Actg2*	NC_000072	Actin, gamma 2, smooth muscle, enteric	0.25	0.1732	1.77	0.0739
*Col4a3*	NC_000067	Procollagen, type IV, alpha 3	2.63	0.0352	3.09	0.0204
*Mmp9*	NC_000068	Matrix metallopeptidase 9	2.41	0.0052	0.88	0.605
*Tgfb-1*	NC_000073	Transforming growth factor beta-1	0.64	0.1862	0.7	0.1773
*Gapdh*	NC_000072	Glyceraldehyde-3-phosphate dehydrogenase	1.6	0.0089	1.98	0.0056

RNA was extracted from dermal fibroblasts cocultured with MSCs for 36 hours. Gene expression levels were measured. Fold changes in mRNA levels were determined by dividing gene expression levels of fibroblasts cocultured with mesenchymal stem cells by gene expression levels of fibroblasts cocultured with control inserts. Genes in the table demonstrated consistent and statistically significant fold changes (*p* < 0.05) in response to MSCs. All changes in mRNA levels were statistically significant and reproduced in three independent experiments

*KO* knock out, *MSC* mesenchymal stem cell, *WT* wild type

### Cotransplantation of MSCs and fibroblasts in the hydrogel matrix system modulates immune responses within wounds

**Fig. 3:**
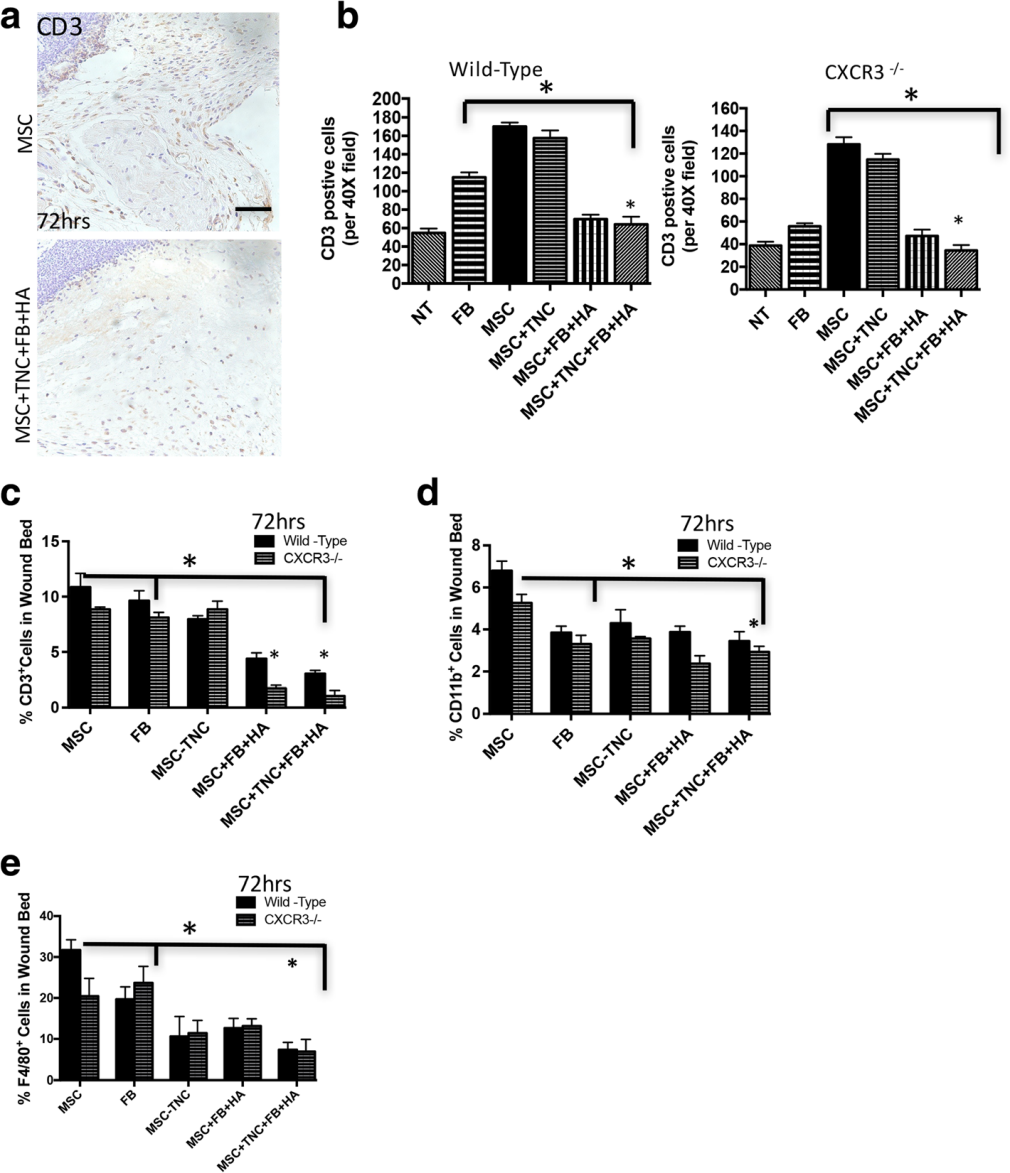
MSCs–fibroblasts aid in reducing immune response and rejection. **a** Immunohistochemistry micrographs of matrix ± MSCs, TNC, fibroblasts (FB), and HA were probed for CD3 to assess inflammatory infiltration in the transplant environment after 72 hours. Shown is mean ± s.e.m. with MSCs + TNC + FB + HA being statistically different from MSCs alone. **b** Quantification of CD3^+^ cells via counting of sections from **a**, with examination of at least four fields per mouse. **c–e** FACS analysis for wound CD3 (T cells), CD11b (pan-leukocytes), and F4/80 (macrophages) in an assessment of wound bed inflammatory infiltrate 72 hours post transplantation, quantified by FlowJo software. *p < 0.05, **p < 0.01, compared to control. Data and images from a representative experiment of three trials, with at least *n* = 3 per experiment per condition, each with a different cell isolate. HA hyaluronic acid, MSC mesenchymal stem cells, NT no treatment, TNC tenascin-C

Excessive or persistent immune responses are a major barrier to effective cellular transplantation, often destroying the transplanted cells and causing additional host tissue damage by an adaptive immune response. In order to test initial rejection response, our xenogeneic–allogeneic MSC–fibroblast hydrogel matrix system was assessed for the infiltration of CD3-positive cells 3 days post wounding during the height of the initial cell influx. We found that the MSCs + fibroblasts encapsulated in the TNC–hydrogel matrix system provided a relatively less immunoreactive (less inflammatory) microenvironment as shown by reduced infiltration of CD3-positive cells when compared to MSC transplant alone, regardless of CXCR3 expression by fibroblasts (Fig. 3a, b). FACS analysis of the total wound bed confirmed the immunoprotective effect of the MSC + fibroblast TNC–hydrogel matrix system treatment. There was a significant reduction of CD3-positive and CD11b-positive cells 72 hours post transplantation (Fig. 3c, d). Interestingly, the CXCR3^–/–^ mice displayed a greater reduction in CD3-positive, CD11b, F4/80-positive wound cells 72 hours post transplantation of the MSC + fibroblast hydrogel matrix than wild-type mice (Fig. 3c–f). The presence or absence of CXCR3 was confirmed by CD183 in both wild-type and CXCR3^–/–^ mice.

### Cotransplantation of MSCs and fibroblasts prevents excessive scarring and phenotypic deficits in wounds

**Fig. 4:**
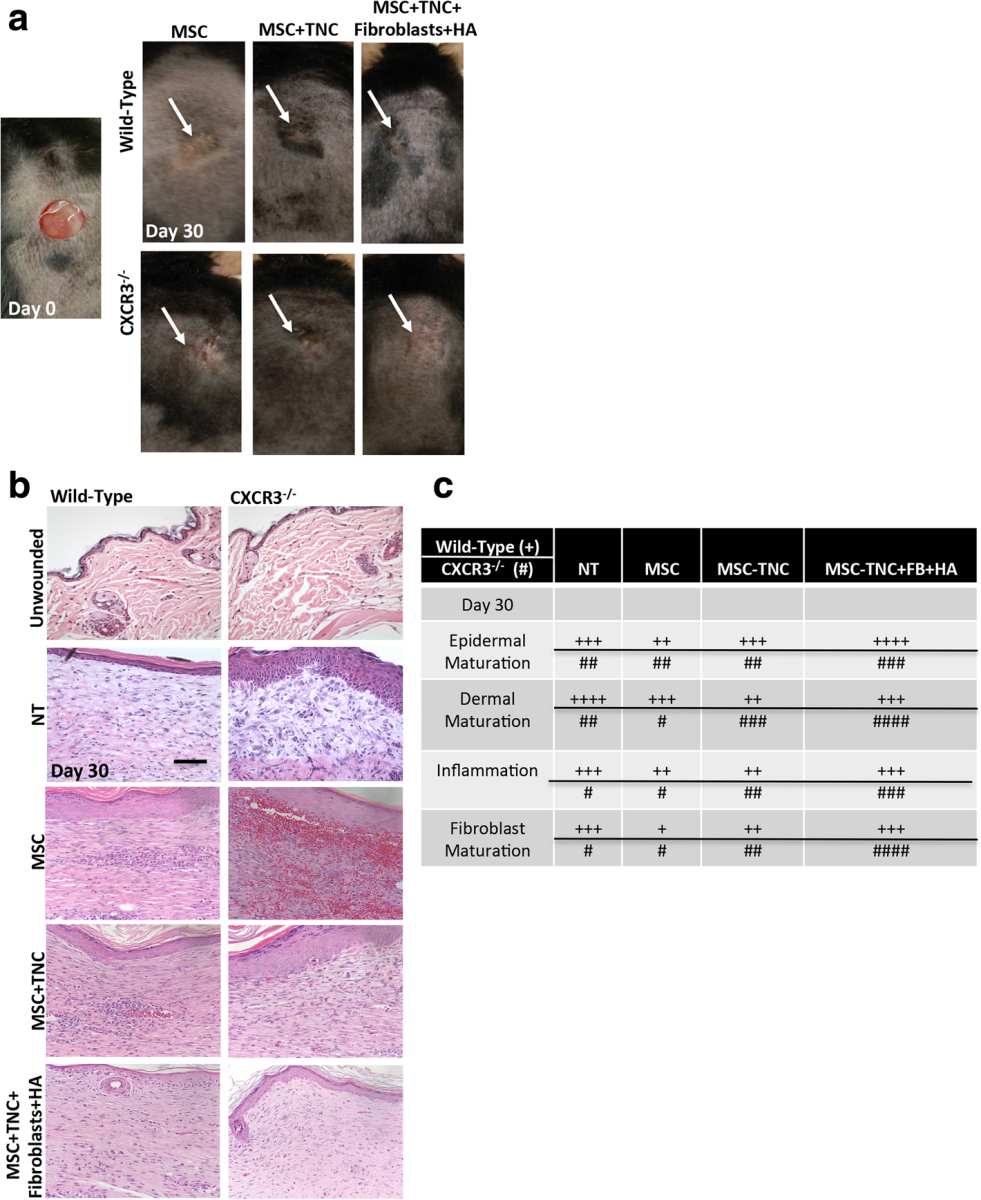
In-vivo analysis of MSC + fibroblast hydrogel matrix system. **a** Representative photographs of full-thickness wounds 30 days post transplantation and with MSCs, MSCs + TNC, and/or MSCs + TNC/FB with HA treatments. **b** H&E micrographs evaluated histologically for inflammation, and maturation of the dermis and epidermis. **c** Quantitative histological assessments of each wound at all time points by a blinded veterinary pathologist (day 30 shown). **+** Wild-type mice, # CXCR3^–/–^. Measurements made on a scale of 0–4 compared to unwounded within each genotype to determine scoring averages. Scale is 0–4 with +/# = 1. *p < 0.05, compared to unwounded genotype. Histological sections and data from a representative at least three mice across three experiments. Original magnifications, ×400. HA hyaluronic acid, MSC mesenchymal stem cells, NT no treatment, TNC tenascin-C

We have published previously that mice lacking CXCR3 present a visible scar, a thickened epidermis, and a disorganized and hypercellular dermis along with persistent and excessive vascularity [23] that leads to a disorganized matrix and, eventually, a hypertrophic scar [19]. Here, we explored the ability of MSCs + fibroblasts encapsulated in the collagen–tenascin-C hydrogel matrix system to improve wound outcomes in CXCR3-deficient mice. Full-thickness excisional wounds on the mouse dorsum were created and followed for 30 days. Gross analysis of these wounds shows that the abnormal healing patterns in CXCR3^–/–^ were improved by treatment with the MSCs + fibroblasts at 30 days post transplantation (Fig. 4a). Histological evaluation of wounds confirmed that the MSC + fibroblast hydrogel matrix system treatment improved healing as shown by limitation of the excessive epidermal cell layers, hyperkeratinization, excessive fibroblasts, and collagen as well as the persistent chronic inflammation seen in the CXCR3-deficient mice when compared to the no treatment (NT) control (Fig. 4b). A deeper histological assessment was performed by a trained veterinary pathologist. Qualitative assessments show that the hydrogel matrix system remarkably enhanced the healing outcomes in CXCR3^–/–^ mice compared to nontreated CXCR3^–/–^ mice. Specifically, CXCR3^–/–^ wound outcomes were enriched in epidermal and dermal maturation and fibroblast infiltration and reactivity as well as less chronic inflammation (presence of plasma and monocytic cells) that is characteristic of CXCR3^–/–^ mice 30 days post transplantation. Additionally, the CXCR3^–/–^ hydrogel matrix system-treated wounds closely resembled normal wild-type healing at 30 days post transplantation. To note, there was a slight improvement of overall epidermal maturation in wild-type mice (Fig. 4c). Taken together these data reveal the dominance and effectiveness of the MSCs + fibroblasts encapsulated in the collagen–tenascin-C incorporated hydrogel matrix system over the dysfunctional healing in CXCR3-deficient mice.

### MSCs augment fibroblast ECM deposition to enhance healing outcomes

**Fig. 5:**
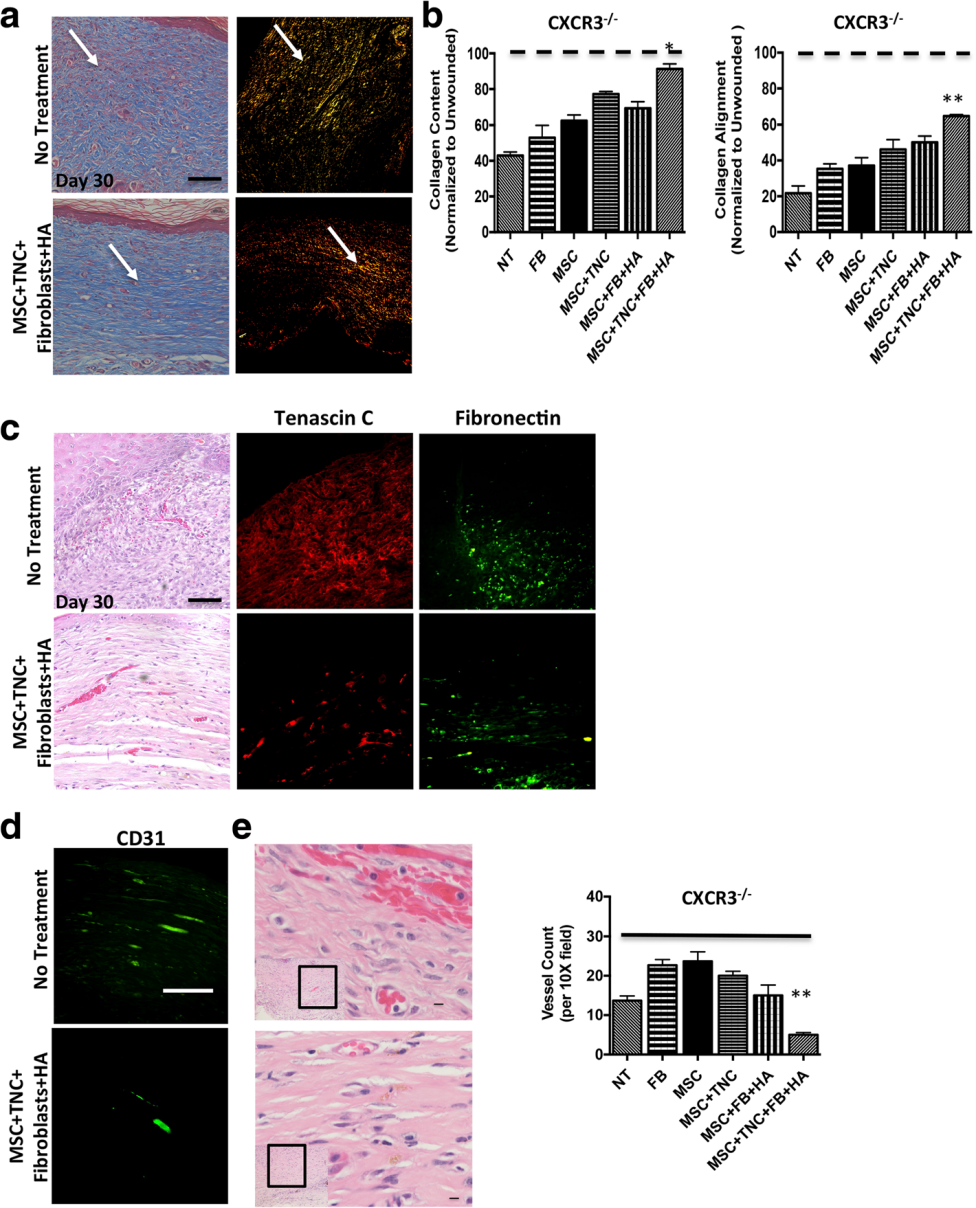
MSC + fibroblast hydrogel matrix system alters ECM remodeling outcomes. **a** Masson’s Trichrome staining used to detect collagen being produced, while Picrosirius Red staining used to detect appropriately aligned collagen bundles. Wounds of CXCR3^–/–^ mice showed distinct patterns of collagen remodeling improvement when treated with MSC + TNC/fibroblast with HA treatments versus no treatment. **b** Wound collagen quantification of both Masson’s Trichrome and Picrosirius Red staining was done by MetaMorph imaging software. CXCR3^–/–^ mice wounds treated with MSCs + TNC/fibroblasts with HA showed a more organized dermal matrix, longer and thicker collagen fibers, and a mature scar, as shown by arrows (determined by total integrated birefringence compared with contralateral unwounded skin) versus that of the NT. **c** Immunohistochemistry of matrix proteins tenascin-C and fibronectin were stained in tissue at 30 days post transplantation. **d, e** Angiogenesis was assessed by immunohistochemistry using CD31 marker and histologically by H&E staining. Vessels were quantified by positive stain counts. Original magnifications: **a, c** × 400, **d** × 200, H&E × 100 and × 600. *p < 0.05, **p < 0.05. Images, histological sections, and data shown from a representative at least three mice across three experiments. FB fibroblasts, HA hyaluronic acid, MSC mesenchymal stem cells, NT no treatment, TNC tenascin-C (Color figure online)

A major function of fibroplasia after wounding is to regenerate the collagen matrix of the dermis. We have found that in the absence of CXCR3 signaling, dermal wound repair results in an overproduction of collagen content yet a lack of collagen alignment and maturation, leading to a weak and immature wound [19, 22]. We assessed the efficacy of the MSCs–fibroblasts encapsulated in the collagen–tenascin-C incorporated hydrogel matrix system to improve the matrix dermal remodeling in CXCR3-deficient mice. Masson’s Trichrome staining shows the MSCs + fibroblasts encapsulated in the collagen–tenascin-C incorporated hydrogel matrix system improved collagen production in CXCR3-deficient mice, to the point of resembling that of wild-type mice and a similar level of collagen to unwounded skin with a denser and more organized granulation tissue (Fig. 5a, b). Picrosirius Red staining, which detects appropriately aligned collagen bundles, shows that the hydrogel matrix system produced more mature aligned fibers 30 days post transplantation compared to the immature fibers and scars that fail to provide strength in the absence of CXCR3 with no treatment (Fig. 5a, b).

The imbalances in expression of key ECM proteins can result in pathological fibrosis. We found that fibrosing wounds have persistent matrix protein turnover, resulting in prolonged turnover of the matrix and continuous remodeling, which are hallmark features of excessive scarring. CXCR3-deficient mice heal more slowly and with a continuous matrix remodeling phenotype [19]. The hydrogel matrix system corrected the persistence in matrix turnover as evidenced by reduced tenascin-C and fibronectin staining 30 days post transplantation in the MSC–fibroblast-treated wounds (Fig. 5c).

The CXCR3^–/–^ phenotype results in persistence of the healing-associated hypervascularity even into the late remodeling/regenerative phase post transplantation, whereas a paucivascular dermis reappears in physiologic healing [19]. This proangiogenic state was greatly reduced after CXCR3^–/–^ mice were treated with the MSC–fibroblast hydrogel matrix system 30 days post transplantation (Fig. 5d, e). These data suggest that there is a mechanism by which the MSC–fibroblast hydrogel matrix system can regulate production and turnover of matrix components, which modulates the angiogenic response to control cellular content during wound repair.

## Discussion

Healing is the complex and dynamic process of restoration of anatomical continuity and function. Following tissue injury there are four responses [21]: regeneration, which is exact replacement as seen in fetal healing; normal repair, which reestablishes equilibrium between scar formation and remodeling; deficient healing, which is the pathological response that occurs when there is insufficient deposition of connective tissue matrix; and excessive healing, which is the pathological response that results in overabundant deposition of connective tissue matrix. The fibroblast has the pivotal job of responding to wound healing by proliferating and chemotaxing into the site of tissue injury to produce the largest part of the mature matrix. Fibroblasts additionally serve key roles in inflammation modulation, immune cell recruitment, and aiding endothelial cells in facilitating angiogenesis [29]. As such, fibroblasts have been studied as cellular targets and therapies to improve the pathological response to injury. However, currently the single target approaches for the treatment of excessive healing have been less then optimal mostly due to the numerous factors at play, including the native environmental cues. As such, simple transplantation of normal fibroblasts into the pathological wound has not yielded the net effect of multiple stimuli that recapitulate the environment in human tissues needed to reestablish equilibrium between remodeling and scar formation. This leads to the critical question of whether the transplanted fibroblasts are responding to the pathological cues of the microenvironment and are unable to promote a change toward normal repair.

MSCs have also been proposed as therapies for lost tissues, due to their potential to differentiate into multiple cell types and form the missing tissues [30]. While preclinical models have been promising, the inability to find these cells incorporated into the repaired tissue has led to questions of whether the improvements are due to production of bioactive factors and modulation of immune/inflammatory responses or a combination thereof [15]. Thus, we proposed that transplantation of MSCs + fibroblasts could educate the wound microenvironment by affecting the mechanisms underlying the tissue/injury diversity to normalize aberrant healing.

In this study, we asked whether bone marrow-derived MSCs delivered along with fibroblasts would improve dermal healing and reduce scarring in a murine hypertrophic scarring model. CXCR3 deficiency in murine skin has been well established as an excessive scarring murine model [19, 21, 23]; additionally, the CXCR3 ligand IP-9AS transgenic murine model results in delayed healing and scar formation in the upper dermis [24]. While we recognize that there are other models of scarring [5], these models are more complex in their mechanism of healing or mechanical induction of scarring. Furthermore, it must be acknowledged that animal models of scarring only approach the human situation but fail to truly recapitulate it [31]. As such, we deemed the CXCR3-deficient murine model well suited for this initial line of investigation. Although outside of this current manuscript, a closer analysis is under investigation to determine the paracrine and juxtacrine effects of MSCs on fibroblast activity. We have begun to probe the potential mechanism involved in the therapeutic effect of MSC + fibroblast treatment.

To test the wound healing effects of MSCs + fibroblasts, an optimal delivery method for the cells was required. This was especially important given that MSCs which have been expanded ex vivo and delivered at sites of injury do not survive for long periods. Previous studies have indicated that, following transplantation, engrafted MSCs are rapidly lost within the first few days [28]. However, in the presence of EGF-receptor signaling from the cell membrane, due to tethered EGF or collagen–tenascin-C, xenotransplanted MSCs can survive and release bioactive factors for 2–3 weeks [18, 32]. This survival reflects our earlier work in vitro, in which we found that the EGF-like repeats in tenascin-C protect MSCs and fibroblasts from induced cell death [18]. Thus, these cells may create a positive effect on the local milieu, although the cells eventually undergo cell death as they are recognized as foreign [18, 32]. To extend the lifespan of MSCs as well as the delivered fibroblasts, this study utilized a hydrogel containing collagen and tenascin-C. Tenascin-C was chosen due to its presence solely in regenerating and not mature or healed skin, and the fact that it contains EGF-like repeats which binds and signals via the epidermal growth factor receptor on MSCs [33]. We have shown previously that a combination of these two matrices improves MSC survival in the face of proinflammatory cytokine challenges [18].

Parenthetically, we have used xenogeneic human MSCs for two reasons: as an extreme example of allotransplant with eventual rejection that would be the norm in supporting healing in debilitated patients, and due to the fact that primary murine MSCs often produce fibrosarcomas when implanted into skin wounds [34]. Still, the MSCs survive sufficiently long to impact the fibroblast and matrix aspect of wound healing. We have used allogeneic fibroblasts in this study, which may be especially beneficial in acute situations and those in which the patient has comorbidities that affect stem cell reserves or functioning (including but not limited to aged and metabolic syndromes). The use of immortalized human stem cells is obviously not compatible with transition to clinical implementation. However, this initial experimental series requires consistency of stem cells to determine the role of matricellular molecule promotion of survival in altering the milieu for wound healing. Future studies will examine primary stem cells as the additive.

The collagen–tenascin-C matrix improved survival of MSCs and fibroblasts; both in vitro in the face of serum deprivation and proinflammatory challenge, and in vivo once implanted onto murine excisional wounds. The matrix also allowed the MSCs to positively modulate the implanted fibroblasts. MSCs were found to not only make the fibroblasts more migratory and proliferative; they also led to fewer inflammatory cells in the wound. More interestingly, the combined cell treatment overcame the excessive scarring that follows the healing of wounds in CXCR3-deficient mice. In the context of scarring, the implanted MSCs prevented hyperkeratinization, as well as excessive matrix deposition by fibroblasts. Using the encapsulated MSC–fibroblast hydrogel matrix in CXCR3-deficient mice we determined that fibroblasts could overcome endogenous deficiencies and generate a more physiological dermal tissue. Furthermore, this xeno/allotransplantation into wounds of the CXCR3-deficient scarring model revealed an increase in survival and reduced apoptosis of fibroblasts, enhanced healing response, correction of matrix regeneration, and reduced inflammation during the first 30 days. Changes in matrix and soluble signals were also examined. These data suggest in-situ matrix composition and mature matrix generation regulates scarring. At a macroscopic level we find that our cell-therapy construct heals wounds more efficiently compared to MSC treatment or fibroblast treatment alone.

All of these findings support a model in which the ECM, produced by the resident cells, dictates the wound outcome [21]. In such a model the cells respond to the matrix as to whether it is in a tissue replacement/regenerative or a wound resolution/quiescent state. This would act dominantly over the cell intrinsic defects. In this model of scarring, the lack of CXCR3 signaling hinders the transition from tissue replacement to resolution so that the fibroblasts continue to produce immature matrix, leading to hypertrophic scar formation in a feed-forward manner. The MSC + fibroblast transplant, provided sufficient time by the tenascin-C survival signals, counters this matrix defect and can generate sufficient resolution-state matrix to now direct even the resident cells toward quiescence to avoid excess tissue build-up.

## Conclusions

This mechanistic model has implications for human wounds of many types in suggesting that healing approaches must correct aberrant matrix composition and signaling to then modify the phenotypes of the resident cells toward healing. We would argue that unless such correction is accomplished, the underlying pathologies will remain and treatments will ultimately fail. The presented data provide insight into cellular therapies as a valuable method for antifibrotic treatment and demonstrate the ability for a long-term impact via matrix modulation and cellular education. In conclusion, unfortunately, as no animal models truly capture the full spectrum of human wound pathologies (whether chronic nonhealing or excessive scarring) [31], this will need to be demonstrated in clinical trials.

## Abbreviations

Col I: Collagen I
ECM: Extracellular matrix
EGF: Epidermal growth factor
HA: Hyaluronic acid
MSC: Mesenchymal stem cell
TNC: Tenascin-C
